# Expression of Thioredoxin/Thioredoxin Reductase System Genes in Aphid-Challenged Maize Seedlings

**DOI:** 10.3390/ijms21176296

**Published:** 2020-08-31

**Authors:** Hubert Sytykiewicz, Iwona Łukasik, Sylwia Goławska, Iwona Sprawka, Artur Goławski, Julia Sławianowska, Katarzyna Kmieć

**Affiliations:** 1 Institute of Biological Sciences, Faculty of Exact and Natural Sciences, Siedlce University of Natural Sciences and Humanities, 14 Prusa St., 08-110 Siedlce, Poland; iwona.lukasik@uph.edu.pl (I.Ł.); sylwia.golawska@uph.edu.pl (S.G.); iwona.sprawka@uph.edu.pl (I.S.); artur.golawski@uph.edu.pl (A.G.); js178@stud.uph.edu.pl (J.S.); 2 Department of Plant Protection, Faculty of Horticulture and Landscape Architecture, University of Life Sciences in Lublin, 7 Leszczyńskiego St., 20-069 Lublin, Poland; katarzyna.kmiec@up.lublin.pl

**Keywords:** maize, *Rhopalosiphum padi*, *Metopolophium dirhodum*, gene expression, thioredoxin, thioredoxin reductase

## Abstract

Thioredoxins (Trxs) and thioredoxin reductases (TrxRs) encompass a highly complex network involved in sustaining thiol-based redox homeostasis in plant tissues. The purpose of the study was to gain a new insight into transcriptional reprogramming of the several genes involved in functioning of Trx/TrxR system in maize (*Zea mays* L.) seedlings, exposed to the bird cherry-oat aphid (*Rhopalosiphum padi* L.) or the rose-grass aphid (*Metopolophium dirhodum* Walk.) infestation. The biotests were performed on two maize genotypes (susceptible Złota Karłowa and relatively resistant Waza). The application of real-time qRT-PCR technique allowed to identify a molecular mechanism triggered in more resistant maize plants, linked to upregulation of thioredoxins-encoding genes (*Trx-f*, *Trx-h*, *Trx-m*, *Trx-x*) and thioredoxin reductase genes (*Ftr1*, *Trxr2*). Significant enhancement of TrxR activity in aphid-infested Waza seedlings was also demonstrated. Furthermore, we used an electrical penetration graph (EPG) recordings of *M. dirhodum* stylet activities in seedlings of the two studied maize varieties. Duration of phloem phase (E1 and E2 models) of rose-grass aphids was about three times longer while feeding in Waza plants, compared to Złota Karłowa cv. The role of activation of Trx/TrxR system in maintaining redox balance and counteracting oxidative-induced damages of macromolecules in aphid-stressed maize plants is discussed.

## 1. Introduction

Plant thioredoxins (Trxs) are a diverse group of oxidoreductases involved in the cleavage of a disulfide bond between the cysteine residues in the target proteins [1,2]. Thioredoxins are localized at different cellular compartments (e.g., chloroplasts, mitochondria, peroxisomes, nucleus, cytosol, plasma membrane, extracellular matrix) and they may be classified into several groups: trx *f*-, *h*-, *m*-, *o*-, *x*-, *y*-, and *z*-type) [3,4,5]. A wide range of trx isoforms enables integration of genes’ expression and metabolic pathways, regulation variety of physiological processes (e.g., flowering, seed germination, seedling growth) as well as stress tolerance and defense responses to environmental stimuli [3,6,7]. The oxidized thioredoxins may undergo a reversible reduction reaction catalyzed by thioredoxin reductases (TrxRs). In higher plants, two Trx/TrxR systems have been found; one of them is linked to ferredoxin-thioredoxin reductase (FTR; EC 1.8.7.2), and the second one is associated with NADPH-dependent thioredoxin reductase (TrxR; EC 1.8.1.9) [8,9]. The Trx/TrxR systems play a pivotal role in restoring intracellular redox homeostasis during both physiological state of the cells and under exposure to environmental stresses, thus limiting oxidative-associated damages to crucial macromolecules in the plants [10,11]. However, there is scarce information regarding transcriptional regulation of the genes encoding thioredoxins and thioredoxin reductases in plant tissues [12]. 

Maize (*Zea mays* L.) is a commonly used model organism in numerous genetic, biochemical and ecotoxicological studies [13,14]. The arising global acreage of maize crops and the increase in the average daily temperature in spring and summer, contributed to higher density of cereal aphids’ populations infesting these hosts [14,15]. In Poland, the bird cherry-oat aphid (*Rhopalosiphum padi* L.) and the rose-grass aphid (*Metopolophium dirhodum* Walk.) are among the major insect pests infesting the maize plants [15,16]. Therefore, identification of maize cultivars exhibiting resistance toward the aphids as well as uncovering the molecular basis of defense mechanisms induced by these insects are of high importance.

Previously, we revealed that *R. padi* and *M. dirhodum* aphid infestation induced accumulation of superoxide anion radicals and hydrogen peroxide in the maize seedlings, depending on the host and aphid genotypes, duration of infestation period and number of insects per plant [17,18,19,20,21]. Furthermore, we recorded profound increments in the content of several markers of oxidative damages of RNA, proteins and lipids in *R. padi*- or *M. dirhodum*-infested maize seedlings [21,22]. Based on our prior results [21,22] evidencing significant disturbances in the thiol metabolism in the aphid-stressed maize seedlings, we hypothesized that aphid infestation may modulate expression of the genes associated with functioning of Trx/TrxR system and/or total activity of TrxR in the maize plants. However, the involvement of Trx/TrxR system in plants exposed to environmental stimuli, remains largely unknown. Especially, there is no available reports regarding the role of Trx/TrxR system in plant–insect interactions. Hence, the aim of the performed study was to uncover the specific patterns of relative expression of Trx/TrxR system genes in the seedling leaves of Złota Karłowa (susceptible) and Waza (relatively resistant) maize cultivars, infested with *R. padi* and *M. dirhodum* aphids. Furthermore, total activity of TrxR in the aphid-challenged maize plants was monitored. The additional purpose of the study was to evaluate the electrical penetration graph (EPG) recordings to unveil *M. dirhodum* aphids stylet passage throughout the maize foliar tissues as well as to identify the feeding modes of these insects in the leaves of the two examined maize varieties (i.e., Złota Karłowa and Waza). The EPG method enables real-time monitoring of changes in electrical signals (i.e., specific waveforms) that correspond to various feeding/probing behavior of piercing-sucking insects in the host plant tissues [23,24]. Previously [22], we assessed profound differences in EPG recordings of *R. padi* aphids in the seedlings of those two maize genotypes. Hence, comparison the EPG data derived from the two cereal aphid species (*M. dirhodum* and *R. padi*) would extend the knowledge regarding stylet positioning and feeding activities in both susceptible and relatively resistant maize varieties.

## 2. Results

### 2.1. Transcriptional Reprogramming of the Studied Thioredoxins-Encoding Genes in Aphid-Stressed Maize Plants

**Figure 1:**
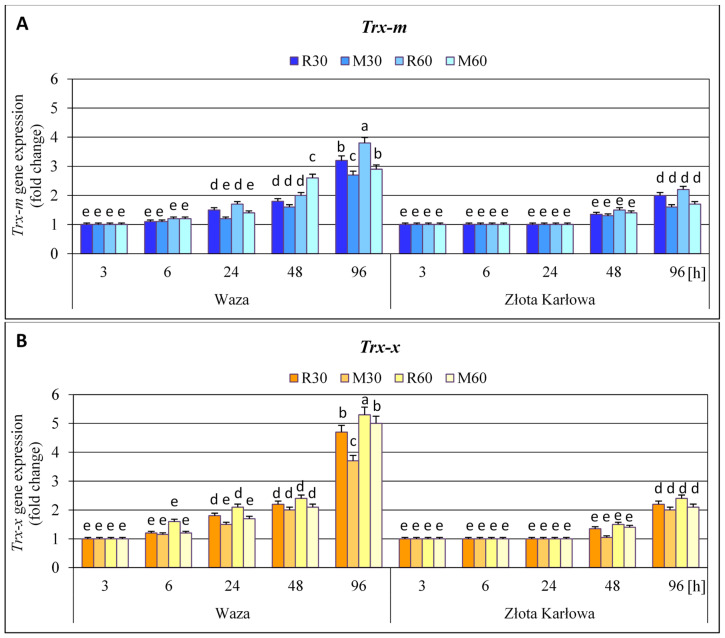
Transcriptional responses of *Trx-m* (**A**) and *Trx-x* (**B**) genes in the aphid-treated maize seedlings (Waza and Złota Karłowa cvs). R30, R60—maize seedlings of a given cultivar infested with 30 and 60 *R. padi* females per plant, respectively; M30, M60—maize seedlings of a given cultivar infested with 30 or 60 *M. dirhodum* females per plant, respectively. Results represent n-fold changes in expression of the examined genes in the aphid-stressed maize seedlings, compared to the non-infested controls. Data were derived from three independent experiments (mean ± SD). Different letters (a, b, c, d, e) above error bars indicate significant differences between the level of *Trx* genes’ expression (*p* < 0.05; post-hoc Tukey’s test).

**Figure 2:**
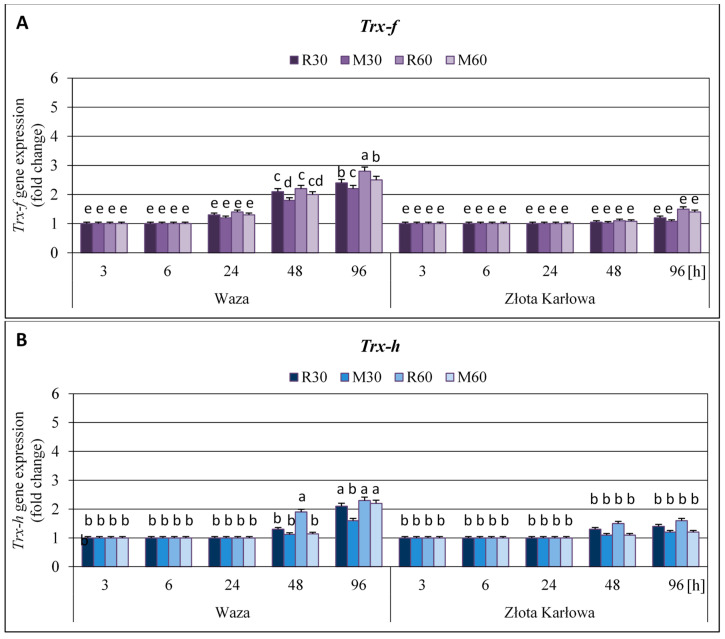
Aphid-stimulated alternations in relative expression of *Trx-f* (**A**) and *Trx-h* (**B**) genes in the leaves of maize seedlings (Waza and Złota Karłowa cvs). R30, R60—maize seedlings of a given cultivar infested with 30 and 60 *R. padi* females per plant, respectively; M30, M60—maize seedlings of a given cultivar infested with 30 or 60 *M. dirhodum* females per plant, respectively. Results represent n-fold changes in expression of the examined genes in the aphid-stressed maize seedlings, compared to the non-infested controls. Data were derived from three independent experiments (mean ± SD). Different letters (a, b, c, d, e) above error bars indicate significant differences between the level of *Trx* genes’ expression (*p* < 0.05; post-hoc Tukey’s test).

It was identified an earlier and stronger upregulation of all the four tested thioredoxins-encoding genes (i.e., *Trx-f*, *Trx-h*, *Trx-m*, and *Trx-x*) in the seedling leaves of more resistant Waza cv., exposed to *R. padi* or *M. dirhodum* aphids’ herbivory in relation to susceptible Złota Karłowa plants (Figure 1 and Figure 2, Supplementary Material: Table S1).

The short-term (3 h) aphid infestation of Waza seedlings did not result in any alternations in expression level of the four studied genes. Similarly, after two consecutive periods of the hemipterans’ herbivory (6 and 24 h) no changes in expression of the *Trx-f* and *Trx-h* genes were detected. On the contrary, amount of the two other transcripts (i.e., *Trx-m* and *Trx-x*) in the aphid-stressed Waza plants increased steadily from 6 to 24 h. Furthermore, the extension of the insects’ infestation period to 48 h led to a significant and gradual increase in the transcriptional activity of all the four examined thioredoxins-encoding genes. Importantly, after 96 h, the highest increments in expression level of the quantified genes occurred in Waza plants (3.7-5.3-, 2.7-3.8-, 2.2-2.8-, and 1.6-2.4-fold elevations in expression of *Trx-x*, *Trx-m*, *Trx-f*, and *Trx-h* genes, respectively), compared to the uninfested control. Besides, the abundance of the four examined transcripts in Złota Karłowa seedlings was unaltered during the first three infestation periods (3, 6 and 24 h), whereas at 48 h only no significant increases (up to 1.5-fold) in expression levels were noted, in relation to the control. Notably, after 96 h, the expression of two tested genes (i.e., *Trx-m* and *Trx-x*) was significantly stimulated by the cereal aphids’ herbivory in Złota Karłowa plants (1.7-2.2- and 2.0-2.4-fold increases, respectively); however, slight and statistically no relevant increases in expression of two other examined genes (i.e., *Trx-f* and *Trx-h*) were found.

In all cases, *R. padi* aphid infestation induced higher elevations in expression of *Trx-f*, *Trx-h*, *Trx-m* and *Trx-x* genes in the seedlings of Złota Karłowa and Waza cvs, compared to the changes caused by *M. dirhodum* infestation. For example, during maximal upregulation of the quantified thioredoxins-encoding genes (at 96 h), *R. padi*-exposed maize seedlings contained 20–90% higher abundance of the four tested thioredoxins-encoding transcripts than *M. dirhodum*-stressed plants. Furthermore, higher number of insects (60 females of a given aphid species per plant) resulted in greater increases in expression level of the examined genes in comparison with lower infestation level (30 aphids per plant).

In addition, the expression level of *trxlp4B* gene encoding the thioredoxin-like protein 4B in foliar tissues of both tested maize cultivars was evaluated. However, the performed analyses did not reveal any significant changes in the transcriptional activity of the studied gene in either Waza or Złota Karłowa maize seedlings infested with *R. padi* or *M. dirhodum* aphids, with respect to the uninfested controls (Supplementary Material: Table S1).

### 2.2. Modulation of Relative Expression of the Thioredoxin Reductase-Encoding Genes (Ftr1 and Trxr2) in Z. mays Seedlings Exposed to the Cereal Aphids’ Herbivory

**Figure 3:**
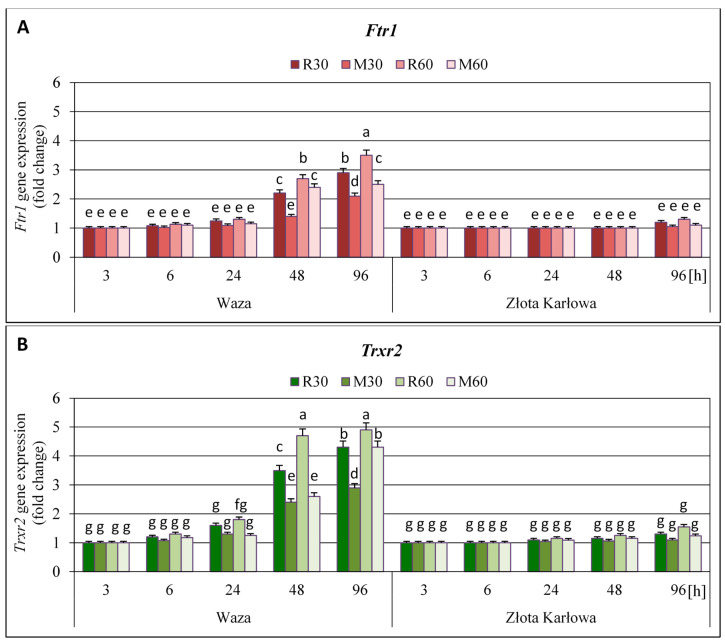
Influence of the cereal aphid infestation on relative expression of *Ftr1* (**A**) and *Trxr2* (**B**) genes in the leaves of maize seedlings (Waza and Złota Karłowa cvs). R30, R60—maize seedlings of a given cultivar infested with 30 and 60 *R. padi* females per plant, respectively; M30, M60—maize seedlings of a given cultivar infested with 30 or 60 *M. dirhodum* females per plant, respectively. Results represent n-fold changes in expression of the examined genes in the aphid-stressed maize seedlings, compared to the non-infested controls. Data were derived from three independent experiments (mean ± SD). Different letters (a, b, c, d, e, f, g) above error bars indicate significant differences between the expression level of the studied genes (*p* < 0.05; post-hoc Tukey’s test).

After 3-h cereal aphids (*R. padi* or *M. dirhodum*) infestation, the amount of *Ftr1* and *Trxr2* transcripts in the seedling leaves of the two examined maize cultivars (i.e., Waza and Złota Karłowa) remained stable, compared to the uninfested controls (Figure 3, Supplementary Material: Table S2). Extending the infestation periods to 6, 24, 48 and 96 h revealed distinct patterns of the transcriptional reprogramming of the two studied thioredoxin reductase genes in Waza and Złota Karłowa plants. The maize seedlings of more resistant Waza cv. infested with *R. padi* or *M. dirhodum* aphids were characterized by profound gradual increments in the abundance of both *Ftr1* and *Trxr2* transcripts. In addition, the transcriptional activity of the *Trxr2* gene was much more stimulated by the cereal aphids’ feeding than the expression of the *Ftr1* gene. It should be underlined that 96-h hemipterans’ infestation caused a maximal upregulation of the two examined thioredoxin reductase genes in Waza plants (2.1-3.5- and 2.8-4.9-fold increases in expression of the *Ftr1* and *Trxr2* genes, respectively). On the contrary, only marginal and insignificant elevations (*p* > 0.05) in expression of the two quantified genes in foliar tissues of Złota Karłowa cv. were recorded (*Ftr1* gene: 3-25% increases at 96 h; *Trxr2* gene: 5–54% increases at 24–96 h). Furthermore, infestation of the maize seedlings with 60 females of *R. padi* or *M. dirhodum* aphids evoked greater elevations in expression of the two tested thioredoxin reductase genes in relation to lower insects’ abundance (30 females of a given aphid species per plant). In general, the stronger effects of *R. padi* females’ infestation on the transcriptional activity of both *Ftr1* and *Trxr2* genes in the maize seedlings of Waza and Złota Karłowa cvs, in comparison with *M. dirhodum*-evoked changes in the respective host plants were demonstrated.

### 2.3. Aphid-Evoked Variations in the Total Activity of Thioredoxin Reductase (TrxR) in Maize Seedlings

**Figure 4:**
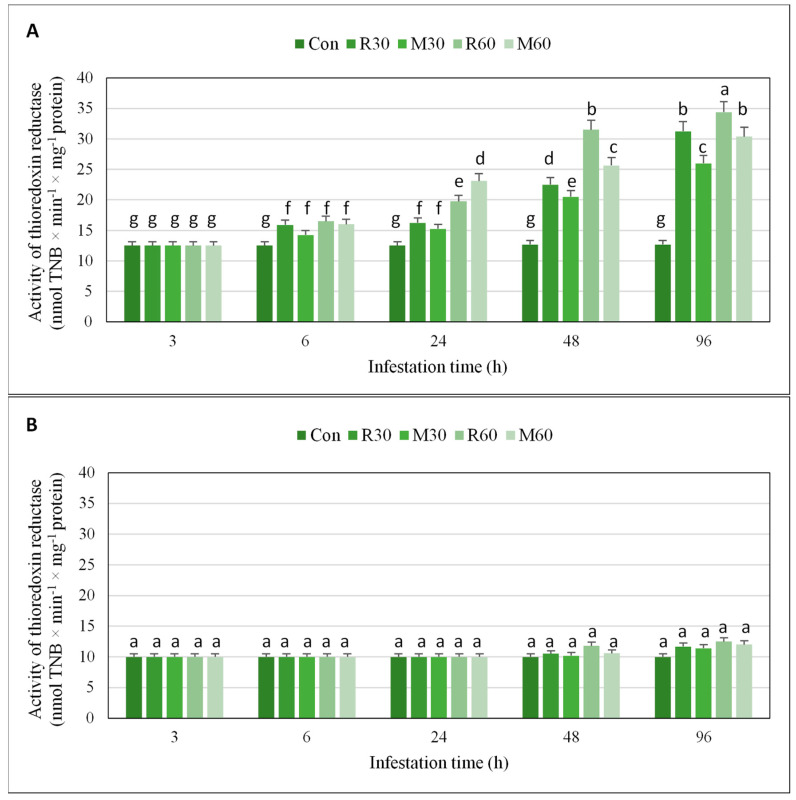
Impact of *Rhopalosiphum padi* and *Metopolophium dirhodum* aphid infestation on the total activity of thioredoxin reductase (TrxR) in the seedlings of two tested maize genotypes. (**A**)—Waza cv.; (**B**)—Złota Karłowa cv.; Con—uninfested (control) plants; R30, R60—maize seedlings of a given cultivar infested with 30 and 60 *R. padi* females per plant, respectively; M30, M60—maize seedlings of a given cultivar infested with 30 or 60 *M. dirhodum* females per plant, respectively. Data were derived from three independent experiments (mean ± SD). Different letters (a, b, c, d, e, f, g) above error bars indicate significant differences between the level of TrxR activity (*p* < 0.05; post-hoc Tukey’s test).

Three-hour feeding of *R. padi* or *M. dirhodum* aphids did not stimulate any changes in TrxR activity in the seedlings of relatively resistant Waza cv. (Figure 4, Supplementary File: Table S2). However, prolongation of the insects’ infestation periods (6, 24, 48, and 96 h) resulted in a gradual increment in the activity of the tested enzyme, compared to the uninfested control plants (27–175% increases in *R. padi*-stressed seedlings; 14–143% increases in *M. dirhodum*-infested plants). Moreover, 30 aphid females per plant induced lower increases in TrxR activity (e.g., 25–150% increases in *R. padi*-infested seedlings, and 14–108% increases in *M. dirhodum*-infested seedlings), compared to higher numbers of aphids, i.e., 60 females per plant (32–175% and 28–143% elevations caused by *R. padi* and *M. dirhodum* infestation, respectively). In almost all cases, *R. padi* aphid infestation induced higher enhancement in TrxR activity in Waza seedlings than *M. dirhodum* herbivory.

Furthermore, the cereal aphid infestation of the susceptible Złota Karłowa seedlings for 3, 6, and 24 h did not cause any alternations in level of the TrxR activity in comparison with the uninfested controls. Nevertheless, after two consecutive periods of the insects feeding (48 and 96 h), slight increases (3–14%, compared to the control) in the TrxR activity were recorded. However, all the elevations in the enzyme activity in the leaves of Złota Karłowa seedlings were not statistically significant.

### 2.4. Electrical Penetration Graph (EPG) Recordings of Feeding Behavior of *M. Dirhodum* Aphids

**Figure 5:**
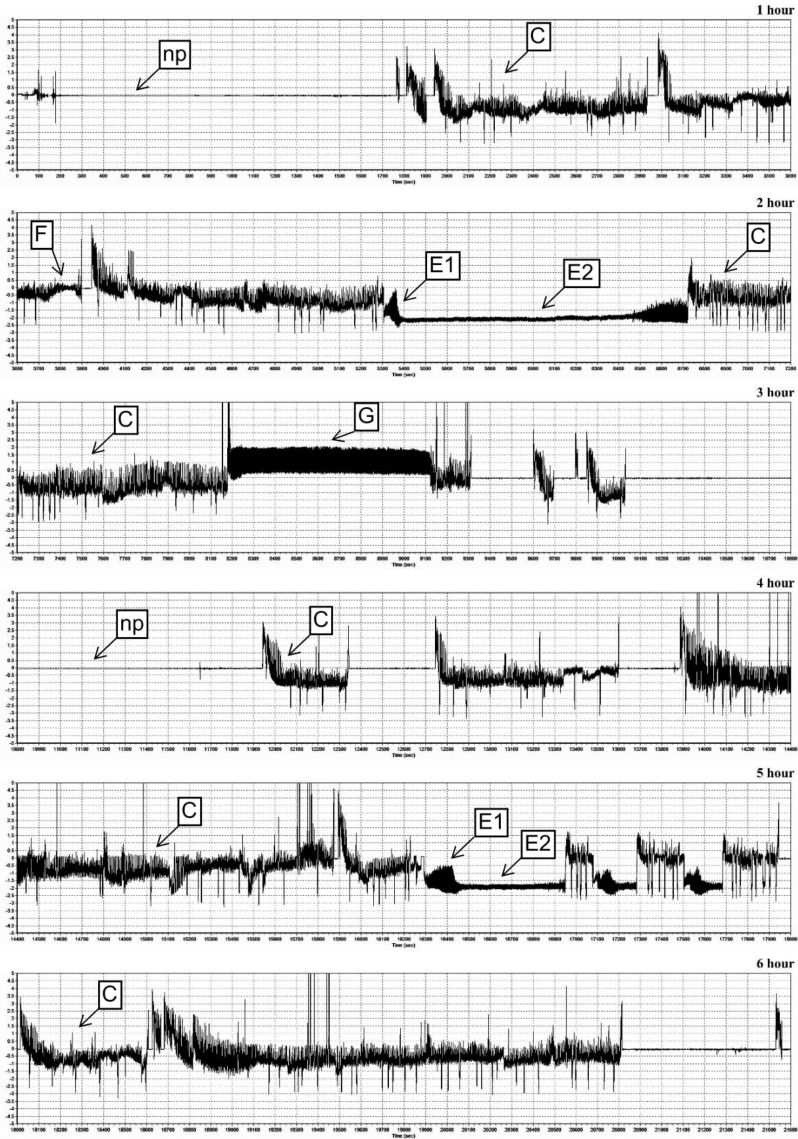
Electrical penetration graph recordings of stylet activities of *M. dirhodum* females in the maize seedling leaves of relatively resistant Waza cv. Y-axis—amplitude (mV); X-axis—time (s); np—non-probing; C—transition of stylet in epidermis and mesophyll cells; E1—salivation into sieve elements; E2—phloem sap ingestion; F—difficulties in stylet penetrations in host plant tissues; G—xylem sap uptake.

**Figure 6:**
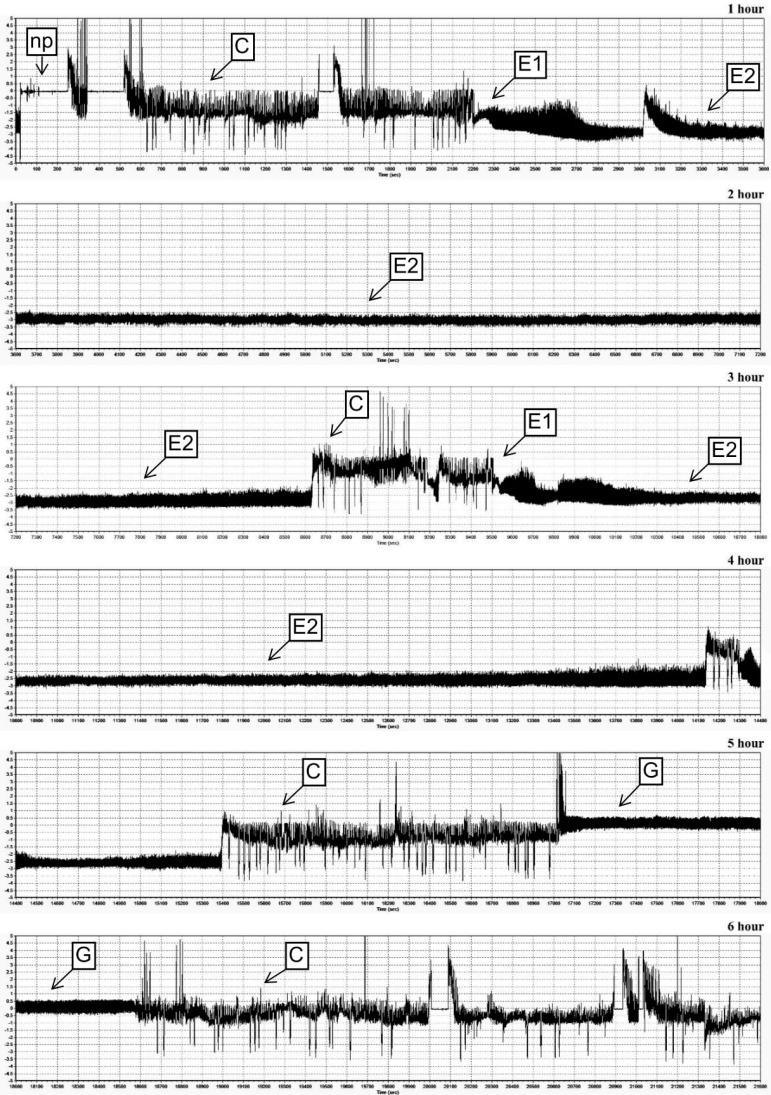
Electrical penetration graph recordings of stylet activities of *M. dirhodum* females in the maize seedling leaves of susceptible Złota Karłowa cv. Y-axis—amplitude (mV); X-axis—time (s); np—non-probing; C—transition of stylet in epidermis and mesophyll cells; E1—salivation into sieve elements; E2—phloem sap ingestion; G—xylem sap uptake.

**Figure 7:**
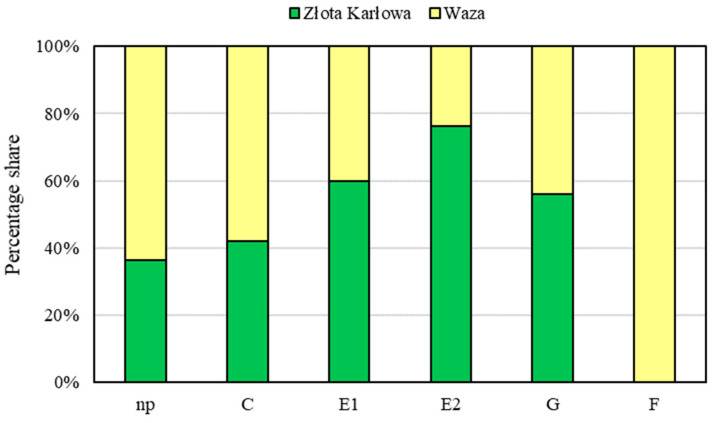
Percentage share of electrical penetration graph (EPG) models during *M. dirhodum* aphids’ feeding in the seedlings leaves of the tested maize cultivars (i.e., Waza and Złota Karłowa); np—non-probing; C—transition of stylet in epidermis and mesophyll cells; E1—salivation into sieve elements; E2—phloem sap ingestion; G—xylem sap uptake; F—difficulties in stylet penetrations in host plant tissues.

Six different EPG waveforms (np, C, E1, E2, G and F) were recorded during infestation of the maize seedlings with *M. dirhodum* females (Figure 5, Figure 6 and Figure 7, Table 1). The first observed waveform was the non-probing phase (np). In this waveform, the aphid’s stylet was outside the plant. The non-probing phase was followed by the stylet penetration (C), indicating transition of the insect stylet through epidermis and mesophyll cells. The next identified waveforms were E1 and E2 models (phloem phase), G (xylem phase) and F (difficulties in stylet penetration throughout the host plant tissues). Pattern E1 indicated salivation into the phloem sieve tubes, pattern E2—ingestion of phloem sap from sieve elements, pattern G—ingestion of xylem sap and pattern F—difficulties in mechanical movements of the aphid’s stylet in plant tissues (Table 1). There were differences in the feeding behavior of *M. dirhodum* aphids fed on the tested susceptible and resistant maize cultivars. The total and average time of penetration of the epidermis and mesophyll were longer on the susceptible Złota Karłowa cv. than on relatively resistant Waza plants. The insects’ feeding on the susceptible seedlings exhibited a prolonged time of phloem sap ingestion. Duration time of E2 waveform was about 117 and 37 min. on the seedlings of susceptible and resistant varieties, respectively. Similarly, the average time of salivation into the sieve elements was substantially longer on the susceptible maize plants. Differences in the percentage share of time duration of EPG waveforms while *M. dirhodum* feeding upon the seedlings of the two maize varieties were found (G-test; G = 17.62, df = 5) (Figure 7). Moreover, the pathway phase (C) represented 43% and 59% of the total recording time on the susceptible and relatively resistant maize plants, respectively. The duration of phloem activities (E1 and E2) of *M. dirhodum* aphids was nearly three times longer when feeding on the susceptible variety, compared to the resistant plants (38% and 14%, respectively), whereas duration of the xylem sap ingestion phase (G) was slightly longer while feeding of the studied insects on Złota Karłowa plants in relation to Waza cv. Importantly, the waveform F (0.5% of the total recording time) was only identified during *M. dirhodum* feeding on the resistant Waza seedlings, and it did not occur in any EPG recordings on the susceptible Złota Karłowa plants.

## 3. Discussion

Previous reports evidenced that the cereal aphid infestation of the maize seedlings was linked to generation of different reactive oxygen species (ROS) in the host tissues [17,18,19,20,21]. The magnitude and time-course dynamics of the oxidative burst in the aphid-exposed maize plants was strongly dependent on the insect species, maize genotype, duration of infestation time and density of aphids per seedling. Sytykiewicz et al. [19] elucidated that seedlings of two maize cultivars (Ambrozja and Tasty Sweet) produced excessive amounts of superoxide anion radicals (O_2_^•−^) in response to *R. padi* or *Sitobion avenae* (F.) aphids infestation, compared to the respective controls. In addition, a higher increase in O_2_^•−^ level in more resistant Ambrozja plants in comparison with susceptible Tasty Sweet seedlings was noted. Superoxide anion radicals are highly reactive and cytotoxic in the plant tissues; therefore, in order to maintain the redox equilibrium state, this ROS form is rapidly converted to less toxic hydrogen peroxide. Furthermore, Sytykiewicz [17] unveiled the principal role of NADPH oxidase and two genes (i.e., *rbohA* and *rbohD*) encoding the specific isoforms of this enzyme in generation of O_2_^•−^ in maize plants subjected to the cereal aphids’ herbivory. In addition, *R. padi*-infested maize seedlings (Ambrozja and Tasty Sweet cvs) treated with diphenyliodonium were characterized by suppressed activity of NADPH oxidase and decreased accumulation of hydrogen peroxide (H_2_O_2_). Łukasik and Goławska [20] also revealed that leaf infestation of Ambrozja and Płomyk maize genotypes with *M. dirhodum* aphids induced overproduction of O_2_^•−^ and H_2_O_2_ in relation to the uninfested controls, and the maximal increase in level of these ROS forms at 24–48 h was demonstrated. Additionally, Sytykiewicz [18] uncovered that the maize seedlings exposed to *R. padi* or *S. avenae* aphid infestation had higher content of H_2_O_2_ than the non-infested controls, and the maximal increase occurred at 24 h. It should be underlined that greater accumulation of H_2_O_2_ molecules was found in foliar tissues of resistant maize genotypes (Ambrozja, Nana, Touran, Waza) in comparison with susceptible varieties (Tasty Sweet, Złota Karłowa). Short-time increments in ROS level in the host tissues are linked to plant innate immunity responses toward adverse environmental stimuli; however, tolerance of the plant systems to long-term exposure to excessive ROS amounts is essential to maintain the functioning of numerous physiological processes. Our recent studies [20,22] demonstrated a significant increase in level of several biochemical markers of the oxidatively-induced damages of total and mRNAs, proteins and lipids in the aphid-stressed maize plants. Łukasik and Goławska [20] reported an increase in the content of malondialdehyde (MDA; biomarker of lipid peroxidation) in *M. dirhodum*-stressed seedlings of Ambrozja and Płomyk maize cvs, and the highest level of this compound at 48–96 h was recorded. Moreover, the aphid-infested seedlings of Ambrozja cv. (relatively resistant) contained a higher amount of MDA than Płomyk cv. (highly resistant). Similarly, Sytykiewicz et al. [22] elucidated that *R. padi* aphid infestation caused up to two times higher accumulation of MDA in maize leaves of susceptible Złota Karłowa cv., compared to more resistant Waza cv., and the highest increments at 24 h were noted. In addition, at this time point of the aphid infestation occurred the highest degree of the electrolyte leakage (indicating disruption of plasma membrane) in relation to the control. Sytykiewicz et al. [22] also revealed a higher accumulation of protein-bound carbonyl groups (biomarker of oxidative stress) in more susceptible Złota Karłowa cv. infested with *R. padi* females in comparison with relatively resistant Waza seedlings. Furthermore, increased level of the protein carbonylation in *M. dirhodum*-infested maize plants was confirmed by Łukasik and Goławska [20]. According to these authors, the aphid infestation resulted in higher increment in the protein-bound carbonyls content in foliar tissues of moderately resistant maize Ambrozja cv. than in highly resistant Płomyk cv. [20]. Recently, we also reported profound effect of the cereal aphid infestation on the thiol metabolism in the seedlings of several maize cultivars [21,22].

In the current study, we demonstrated that *R. padi* or *M. dirhodum* females’ infestation led to higher increases in expression of the four studied genes (i.e., *Trx-f*, *Trx-h*, *Trx-m*, and *Trx-x*), encoding the respective isoforms of thioredoxins in the leaves of resistant Waza plants, compared to the susceptible Złota Karłowa seedlings. Moreover, stronger effect of *R. padi* apterae feeding than *M. dirhodum* infestation on expression level of the four thioredoxin-encoding genes in the seedlings of both examined maize cultivars was observed. Notably, the aphid-stressed seedlings of relatively resistant Waza cv. were characterized by significant increases in expression of all four thioredoxin genes, whereas cereal aphids herbivory on susceptible Złota Karłowa plants evoked significant upregulation of only two examined genes (i.e., *Trx-m* and *Trx-x*) at 96 h. In addition, we also revealed the highest and progressive elevations in expression of *Trx-m* and *Trx-x* genes in relatively resistant Waza plants, thus evidencing the crucial role of the encoded chloroplastic isoforms of thioredoxins (*m*- and *x*-type) in conferring augmented resistance to *R. padi* or *M. dirhodum* aphid infestation. We also evidenced the significant upregulation of *Ftr1* and *Trxr2* genes in resistant Waza cv. plants only at two time intervals of the aphid infestation (i.e., 48 and 96 h), while no relevant increases in susceptible Złota Karłowa seedlings were observed. However, we identified an earlier and gradual enhancement of TrxR activity in foliar tissues of Waza seedlings (from 6 to 96 h), indicating posttranscriptional regulation mechanism of the enzyme activity. Furthermore, the aphid-stressed Złota Karłowa (susceptible) plants had similar levels of TrxR activity to those in the non-infested controls. In general, *R. padi* females’ feeding caused higher increments in expression of both *Ftr1* and *Trxr2* genes and total activity of the TrxR enzyme in the two tested maize cultivars, compared to *M. dirhodum*-evoked changes.

Major biological functions of plant thioredoxins include posttranslational redox regulation of a wide array of proteins, participation in antioxidative scavenging and signaling networks, overcoming oxidative damages to macromolecules as well as intercellular communication [6,25,26]. Several isoforms of thioredoxins (*f*-, *m*-, *x*-, *y*-, and *z*-type) have been identified in chloroplasts, and their main biological role is associated with modulation of activity of target proteins involved in photosynthesis process [27,28]. It has been also reported that chloroplastic thioredoxins *f*- and *m*-types are involved in regulation of the Calvin–Benson cycle activity. Bohrer et al. [29] evidenced that plastidial thioredoxin genes in all tested organs of *Arabidopsis thaliana* L. plants (i.e., young seedlings, mature leaves, roots, flowers, siliques and seeds) were expressed. The highest abundance of transcripts encoding thioredoxin *m*- and *x*-types in seedlings and mature leaves was recorded; intermediate expression of *TRXf1*, *TRXf2*, and *TRXy2*-genes occurred in the vast majority of the examined organs, whereas the lowest level of *TRXz* transcript in leaves, flowers and siliques was demonstrated [29]. Furthermore, Okegawa et al. [30], using Western-blotting approach, identified accumulation of five types of thioredoxin proteins (i.e., trx-*f*, trx-*m*, trx-*x*, trx-*y*, and trx-*z*) in the leaves of *A. thaliana*. In addition, detailed analyses revealed predominant abundance of thioredoxins *m*- and *f*-type in the chloroplast stroma of foliar tissues of *A. thaliana* [30]. Recently, Sekiguchi et al. [31] documented that two major chloroplastic thioredoxins (*f*- and *m*-type) in *A. thaliana* plants are responsible for light-dependent reduction of CF1-γ subunit of ATP synthase. Additionally, cytosolic thioredoxins *h*-type may also participate in regulation of seed germination process, whereas thioredoxins *f*-type may also be involved in starch accumulation [28,32]. Oxidized thioredoxins undergo reduction reaction catalyzed by two types of thioredoxin reductases (i.e., ferredoxin–thioredoxin reductase and NADPH-dependent thioredoxin reductase) [33,34]. The efficient functioning of Trx/TrxR systems in the plant tissues contributes to maintaining redox balance in the cells under physiological and unfavorable environmental conditions. However, there are no available reports regarding involvement of the Trx/TrxR systems in plant defense reactions to phytophagous insects. 

Jing et al. [9] revealed that osmotic stress induced by high concentration of mannitol led to upregulation of *KcTrxf* gene (encoding chloroplastic thioredoxin *f*-type) in tissues of *Kandelia candel* (L.) Druce. The major function of this thioredoxin is linked to disulfide bonds reduction and maintaining ROS homeostasis in the cells. Additionally, overexpression of *KcTrxf* gene in tobacco plants resulted in elevated tolerance to drought and osmotic stresses, through increased stomatal closure, lower contents of H_2_O_2_ and MDA, and higher integrity of plasmalemma. Importantly, the transgenic tobacco plants overexpressing thioredoxin *f*-type under drought stress conditions enabled sustaining redox homeostasis and had higher ability to regeneration of glutathione (GSH) from its oxidized form [9]. In addition, two atypical maize thioredoxins, ZmTrm2 and ZmTrxh, exhibit chaperone activity, and also participate in shaping resistance toward potyviruses [35,36]. Shi et al. [35] demonstrated an upregulation of *ZmTrm2* gene 10 days after inoculation of the sugarcane mosaic virus (SCMV). These authors postulated that increased biosynthesis of thioredoxin *m*-type downregulated the class I of *beta*-1,3-glucanase (*GluI*) gene, involved in callose formation, and negatively affected replication, accumulation and movement of the SCMV virus in the maize tissues. Moreover, expression of maize *ZmTrm2* gene in the leaves of *Nicotiana tabacum* L. also repressed replication of tobacco vein-banding mosaic virus (TVBMV). Furthermore, Liu et al. [36] revealed that expression level of maize *ZmTrxh* gene (encoding thioredoxin *h*-type) reflected the resistance degree of inoculated maize plants against the SCMV infection. However, atypical maize thioredoxin *h*-type is localized in the cytosol, and it is not able to process a reduction reaction of disulfide bonds because of lack of two canonical cysteine residues in the active site. Additionally, Thormählen and co-workers [37] reported that *Trxf1 Ntrc* double mutants of *A. thaliana* (deficient expression of thioredoxin *f1*-type and NADPH-dependent thioredoxin reductase C, respectively) were characterized by substantial suppression of starch biosynthesis as well as disturbed functioning of the Calvin–Benson cycle and light acclimation. According to these authors, the two thioredoxin systems present in chloroplasts work together in order to regulate the plant growth under diverse light conditions. Richter et al. [6] claimed that redox-control biogenesis of chlorophylls in higher plants is mainly linked to functioning of a wide spectrum of thioredoxins. In addition, *A. thaliana* seedlings with inactivated expression of three *m*-type thioredoxins-encoding genes (i.e., *Trxm1*, *Trxm2*, *Trxm3*) had a lower content of chlorophylls and their metabolites in relation to the wild-type plants [38]. Besides, silencing of these three genes decreased biosynthesis of multitude enzymes involved in biogenesis pathway of chlorophylls (e.g., 5-aminolevulinic acid dehydratase, Mg-chelatase, NADPH-protochlorophyllide oxidoreductase, Mg-protoporphyrin IX methyltransferase /CHLM/). Furthermore, direct interactions of thioredoxins *m*-type and NADPH-dependent thioredoxin reductase C with CHLM proteins were revealed [38,39]. Importantly, Sytykiewicz et al. [40] reported that *R. padi* and *S. avenae* females’ infestation caused a significant decrease in the content of chlorophylls in foliar tissues of several maize genotypes, and the highest depletion in the chlorophyll level occurred in the seedling leaves of susceptible maize cultivars, compared to resistant ones [40]. 

Proteomic analyses performed by Huihui et al. [41] revealed that the seedling leaves of mulberry (*Morus alba* L.) exposed to NaCl stress were characterized by increased biosynthesis of thioredoxin F, thioredoxin O1, thioredoxin-like protein CITRX and thioredoxin-like protein CDSP32 as well as enhanced activity of catalase and enzymes involved in the ascorbic acid-glutathione cycle. As a consequence, formation of superoxide anion radicals and hydrogen peroxide molecules in the stressed plants was efficiently suppressed. On the other hand, the mulberry seedlings subjected to NaHCO_3_ stress were characterized by elevated O_2_^•−^ and H_2_O_2_ amounts, and decreased activity of ferredoxin-thioredoxin reductase (FTR), thioredoxins M4 and F as well as thioredoxin-like protein CDSP32, compared to the non-stressed control, thus indicating a repression of thioredoxin-based antioxidative responses [41].

Aphid stylet passage, saliva excretion and food ingestion in the host tissues cannot be directly investigated, but these activities can be monitored with application of the electrical penetration graph (EPG) technique. This approach is widely used to assess the probing and ingestion behavior of the aphids [42,43,44,45,46]. Analysis of the several parameters derived from the EPG recordings (e.g., frequency, duration and sequence of different waveforms) enables in-depth assessment of behavioral responses of the aphids to the host plant tissues [23,24]. In the current work, the EPG recordings allowed identification of significant differences in probing and feeding behavior of *M. dirhodum* females in the seedlings of Złota Karłowa cv., compared to Waza plants—which was in agreement with previous results reported for infestation of the maize plants with *R. padi* apterae [22]. Moreover, *M. dirhodum* aphids spent most events of activity on walking on the plant surface (np; non-probing phase) and stylet paths (C) in tissues of relatively resistant Waza cv. in relation to Złota Karłowa seedlings. We suggest that the feeding behavior of *M. dirhodum* females was influenced by qualitative and/or quantitative composition of the plant tissues. It has been reported that chemical constituents of the hosts may profoundly affect the aphids’ behavior [22,47,48,49]. In general, these insects spent more time on ingestion of phloem sap, and periods of the stylet movements were shorter while feeding in the plants with a high nutritive quality [50]. In our study, *M. dirhodum* females spent 43% and 59% of the recorded time on penetration epidermis and mesophyll on susceptible (Złota Karłowa) and relatively resistant (Waza) maize plants, respectively. Overall, the rose-grass aphids fed considerably longer on Złota Karłowa seedlings than on Waza cv. Furthermore, there were significant differences in duration of the E1 and E2 phases of *M. dirhodum* aphids’ feeding on seedlings of the two tested maize cultivars. This led to the conclusion that longer time of penetration of aphid stylets in the epidermis and mesophyll and shorter penetration of the sieve elements may be caused by a higher concentration of deterrent chemical factors in the resistant Waza cv. Similarly, our previous study has shown that the bird cherry-oat aphids spent less time on ingestion of phloem sap on more resistant *Z. mays* seedlings (Waza cv.) in comparison with susceptible (Złota Karłowa cv.) plants [22]. Additionally, Goławska et al. [50] observed that the apterous adult pea aphids (*Acyrthosiphon pisum* Harr.) spent more time on ingestion of phloem sap on the alfalfa (*Medicago sativa* L.) plants with low level of saponins, compared to alfalfa Radius cv. that was characterized by high saponin content. Moreover, Zehnder et al. [51] demonstrated that the cowpea aphids (*Aphis craccivora* Koch) spent less time on phloem sap ingestion on more resistant varieties of yellow and narrow-leafed lupines, in relation to the susceptible ones. 

The identified molecular mechanism of substantial enhancement of the Trx/TrxR system in aphid-stressed maize seedlings of resistant (Waza) genotype may contribute to a multi-level reduction of oxidative stress and its detrimental cytotoxic effects in host plant cells. Important aspect of further investigations of maize-aphids interactions should be focused on identification of microRNAs differentially expressed in the seedlings of maize genotypes, exhibiting distinct resistance levels to the insects’ infestation. Regulation of the host genes’ expression by microRNAs may be a pivotal part of complex molecular strategies shaping defense responses of maize cultivars to the cereal aphids’ herbivory.

## 4. Materials and Methods 

### 4.1. Plant Material

The seeds of two examined maize cultivars (i.e., Waza and Złota Karłowa) were purchased from the local grain companies: PNOS S.A. (Ożarów Mazowiecki, Poland) and W. Legutko (Jutrosin, Poland). Selection of the maize cultivars was based on our previously performed biotests, indicating that Waza and Złota Karłowa genotypes were susceptible and relatively resistant to the cereal aphid infestation, respectively [40].

The seeds were surface sterilized, according to the procedure described by Sytykiewicz et al. [22], and next, they were planted individually in plastic pots (10 × 9 cm; diameter × height) filled with the universal garden soil Kronen^®^ (Lasland Sp. zo.o., Grądy, Poland). The seedlings grown in a climate chamber (L16:D8 photoperiod, temperature of 22 °C/16 °C (day/night), 70% relative humidity, 100 μM × m^−2^ × s^−1^ light intensity).

### 4.2. Insects

Parthenogenetic apterae of the bird cherry-oat aphid (*Rhopalosiphum padi* L.) and the rose-grass aphid (*Metopolophium dirhodum* Walk.) were gathered from cereal crops in Siedlce district, Poland (52°09′54″ N; 22°16′17″ E). The aphids were reared for a year on wheat seedlings (*Triticum aestivum* L., Tonacja cv.) in a climate chamber under conditions described above. 

### 4.3. Aphid Infestation Biotests

Insect infestation experiments were conducted as described previously [22]. Fourteen-day-old seedlings of the two examined maize cultivars (i.e., Waza and Złota Karłowa) were artificially infested with 0 (control), 30 or 60 adult parthenogenetic females of *R. padi* or *M. dirhodum* for 0, 3, 6, 24, 48, and 96 h.

### 4.4. Isolation of Total RNA and cDNA Synthesis

Plant material (freshy collected leaves from both insect-infested and control maize seedlings) were homogenized in liquid nitrogen, and 100 mg portions of the powder were used in RNA isolations. Extraction of total RNA from insect-infested and control maize plants was achieved with the use of Spectrum Plant Total RNA Kit (Sigma-Aldrich, Poznań, Poland; catalogue no. STRN50), following the manufacturer’s instructions. In addition, the residual genomic DNA was hydrolyzed using On-Column DNase I Digestion Set (Sigma-Aldrich Poznań, Poland; catalogue no. DNASE70). Quality and quantity of RNA were assessed with the use of an Epoch UV-Vis microplate spectrophotometer (BioTek, Winooski, VT, USA), and only intact RNA samples were further analyzed.

### 4.5. Gene Expression Analyses

Transcriptional responses of the seven studied genes (i.e., *Trx-f*, *Trx-h*, *Trx-m*, *Trx-x*, *Trx-lp4B*, *Ftr1*, *Trxr2*) in foliar tissues of the maize seedlings were measured with application of real-time qRT-PCR technique. The results were normalized to the reference actin-2 gene (*GenBank* accession no. EU952376.1), using *Custom TaqMan Gene Expression Assay* (ID: Zm04008474_g1; Thermo Fisher Scientific Inc., Waltham, MA, USA). Relative expression of the six tested genes (i.e., *Trx-f*, *Trx-h*, *Trx-m*, *Trx-x*, *Trx-lp4B*, *Trxr2*) in the maize seedlings was determined using *TaqMan Gene Expression Assays* (Thermo Fisher Scientific Inc., Waltham, MA, USA) (Table 2), whereas *Ftr1* transcript abundance was quantified using *Custom TaqMan Gene Expression Assay* (Supplementary Material: Table S3). DNA amplification and fluorescent detection was performed using StepOnePlus Real-Time PCR System (fast mode), and the obtained data were analyzed with the StepOnePlus Software v2.3 (Thermo Fisher Scientific Inc., Waltham, MA, USA). The gene expression measurements were performed following the procedure described by Sytykiewicz [52]. The relative expression of the genes was determined by the comparative C_t_ (ΔΔC_t_) method [53], and the results were presented as n-fold changes (±SD) in transcript abundance in the aphid-exposed maize seedlings, compared to the control (aphid-free) plants. Four biological and three technical replications were conducted for each round of real-time qRT-PCR reactions.

### 4.6. Thioredoxin Reductase (TrxR) Activity Assay

Freshly collected leaves of the maize seedlings (Waza and Złota Karłowa cvs) were ground in liquid nitrogen. Total activity of the thioredoxin reductase (TrxR) was determined using the colorimetric Thioredoxin Reductase Assay Kit (Abcam, Cambridge, Great Britain; catalogue no. ab83463), following the manufacturer’s protocol. Portions of the powder (50 mg) were homogenized with 200 μL ice-cold Assay Buffer. Next, the samples were centrifuged at 10,000 × *g* for 15 min. (at 4 °C). The supernatant was collected and used for determination of TrxR activity. Measurement of TrxR activity was based on reduction of DTNB (5, 5′-dithiobis (2-nitrobenzoic) acid to TNB (5-thio-2-nitrobenzoic acid). The yellow color developed was measured at λ = 412 nm, using a microplate UV-Vis spectrophotometer (BioTek, Winooski, VT, USA). One unit (U) of TrxR activity was defined as the amount of enzyme that generates 1.0 μmol of TNB per minute at 25 °C. Total activity of TrxR in the maize samples was expressed as nmol of TNB per minute per milligram of protein. The protein content in the extracts was determined using Lowry method [54].

### 4.7. Electrical Penetration Graph (EPG) Recordings

Feeding behavior of adult wingless females of *M. dirhodum* on the maize plants was evaluated with the use of the electrical penetration graph (EPG) technique, according to the procedure described previously [22]. The aphid and the plant are parts of an electrical circuit that closes when the insect’s stylet is inserted into the host tissues. Specific changes in electrical signals during aphids’ feeding are recorded as EPG waveforms [23,24,50,51]. In the study, the following waveform patterns of the aphids’ feeding activities were monitored: non-probing (np), paths (C; penetration of epidermis and mesophyll), salivation into sieve elements (E1), ingestion of phloem sap (E2), difficulties in aphid stylet penetrations in tissues of the host plant (F), and ingestion of xylem sap (G). The EPG recordings were performed on 14-day-old maize seedlings of two examined maize varieties: Złota Karłowa (susceptible) and Waza (relatively resistant to the tested cereal aphids).

### 4.8. Statistical Analyses 

All data were derived from three independent replicates. Factorial analysis of variance (ANOVA) was used to evaluate the significance of the studied indicators (i.e., maize genotype, aphid species, number of insects, infestation time), and the interactions on relative expression of the seven investigated genes (i.e., *Trx-f*, *Trx-h*, *Trx-m*, *Trx-x*, *Trx-lp4B*, *Ftr1*, and *Trxr2*) and TrxR activity in the aphid-stressed *Z. mays* seedlings. Next, post-hoc Tukey’s test was employed (*p* < 0.05). Significant differences for each EPG waveform between *M. dirhodum* feeding activities on the susceptible and relatively resistant maize cultivars were estimated using *t*-test (*p* < 0.05). The proportion of time devoted to different activities for *M. dirhodum* aphids on maize cultivars was compared using G-test [55]. All analyses were conducted with the use of STATISTICA v.10 software (StatSoft Inc., Kraków, Poland).

## 5. Conclusions

In conclusion, the cereal aphids (*R. padi* or *M. dirhodum*) herbivory stimulated expression of the four thioredoxin-encoding genes (*Trx-f*, *Trx-h*, *Trx-m*, and *Trx-x*) and two thioredoxin reductase genes (*Ftr1*, *Trxr2*) in the maize seedlings of relatively resistant Waza cv. In parallel, significant increase in TrxR activity in the aphid-infested Waza plants was found, thus indicating posttranscriptional regulation of the examined enzyme. The stronger activation of the investigated Trx/TrxR system in the aphid-stressed Waza seedlings may imply higher ability to maintain thiol-based redox equilibrium and counteract oxidative damage to macromolecules in comparison with the susceptible Złota Karłowa cv.

## Figures and Tables

**Table 1:** Stylet activities of *M. dirhodum* aphids during feeding on the seedling leaves of the tested maize cultivars.

EPG Waveforms	EPG Activity			
	Number of Events	Total Duration (min.)	Average Time (min.)	Time to First Probing (min.)
**Złota Karłowa cv. (Susceptible)**				
np	13.85 ± 2.37	43.74 ± 10.68 *	3.32 ± 0.42	n/a
C	20.10 ± 2.69	154.06 ± 7.93 *	7.87 ± 1.06 *	4.16 ± 0.01
E1	6.10 ± 0.47	19.49 ± 6.24	3.50 ± 1.55 *	55.09 ± 9.76
E2	4.35 ± 0.16	116.73 ± 7.39 *	32.31 ± 0.89 *	62.09 ± 9.99
G	2.35 ± 0.15	25.98 ± 4.98	11.47 ± 5.08	141.45 ± 15.88
F	0.00 ± 0.00	0.00 ± 0.00	0.00 ± 0.00	0.00 ± 0.00
**Waza cv. (Relatively Resistant)**				
np	13.55 ± 1.42	74.24 ± 16.55 *	6.57 ± 1.27	n/a
C	22.85 ± 2.21	210.86 ± 7.86 *	9.62 ± 0.39 *	6.42 ± 0.82
E1	7.35 ± 0.95	14.71 ± 4.35	1.83 ± 0.43 *	61.19 ± 10.11
E2	5.05 ± 0.32	37.18 ± 3.69 *	8.93 ± 0.72 *	63.96 ± 10.29
G	2.05 ± 0.02	21.16 ± 1.17	10.23 ± 0.59	121.21 ± 11.23
F	1.60 ± 0.16	1.84 ± 0.52	0.89 ± 0.51	88.74 ± 25.44

Data are presented from 6-h EPG recordings (mean ± SE; *n* = 20). Values for each EPG waveform within a column marked by asterisk are significantly different between the maize cultivars (*t*-test; *p* < 0.05); np—non-probing; C—transition of the insect stylets through epidermis and mesophyll cells; E1—salivation into sieve elements; E2—phloem sap ingestion; G—xylem sap uptake; F—difficulties in stylet penetrations in tissues of the host plant; n/a—not applicable.

**Table 2:** The list of the quantified maize genes and *TaqMan Gene Expression Assays* ^1^.

Gene	*GenBank* Accession No.	*GenBank* Reference Sequence	Assay ID	Gene ID
*Trx-f*(thioredoxin *f*-type)	BT036952.1	NM_001142977.1	Zm03994181_g1	LOC100216557
*Trx-h*(thioredoxin *h*-type)	EU953886.1	NM_001153720.2	Zm04083851_s1	LOC100280800
*Trx-m* (thioredoxin *m*-type)	EU965627.1	NM_001157280.1	Zm04079742_s1	LOC100284385
*Trx-x* (thioredoxin *x*-type)	EU954216.1	NM_001153772.1	Zm04081670_s1	LOC100280852
*Trx-lp4B* (thioredoxin-like protein 4B)	EU954451.1	NM_001153812.1	Zm04013946_m1	LOC100280892
*Trxr2* (thioredoxin reductase 2)	EU966898.1	NM_001156943.1	Zm04083908_g1	LOC100284045

^1^*TaqMan Gene Expression Assays* (Thermo Fisher Scientific Inc., Waltham, MA, USA).

## Supplementary Materials

The following are available online at https://www.mdpi.com/1422-0067/21/17/6296/s1, Table S1: Factorial ANOVA results of the tested parameters (maize genotype, aphid species, number of aphids, infestation time) and the interactions on relative expression of the examined thioredoxins-encoding genes (*Trx-f*, *Trx-h*, *Trx-m*, *Trx-x*, *Trx-lp4B*) in the maize seedlings; Table S2: Factorial ANOVA results of the tested parameters (maize genotype, aphid species, number of aphids, infestation time) and the interactions on the relative expression of the thioredoxin reductase genes (*Ftr1*, *Trxr2*), and the total activity of thioredoxin reductase (TrxR) in the maize seedlings; Table S3: List of primers and *TaqMan* fluorescent probe for real-time qRT-PCR quantification of *Ftr1* maize gene.
