# Supplementary material for: Expression of Thioredoxin/Thioredoxin Reductase System Genes in Aphid-Challenged Maize Seedlings
Source: Int J Mol Sci. 2020 Aug 31;21(17):6296. doi: 10.3390/ijms21176296 (PMC7503728; doi:10.3390/ijms21176296)
Supplement: Supplementary file 1 [file ijms-21-06296-s001.pdf]

**Table S1.** Factorial ANOVA results of the tested parameters (maize genotype, aphid species, number of aphids, infestation time) and the interactions on relative expression of the examined thioredoxins-encoding genes (*Trx-f*, *Trx-h*, *Trx-m*, *Trx-x*, *Trx-lp4B*) in the maize seedlings.

| Tested Effects and Interactions | *Trx-f* | *Trx-h* | *Trx-m* | *Trx-x* | *Trx-lp4B* |
|---|---|---|---|---|---|
| Maize genotype (G) | $F_{1, 96}$ = 1,615 (***) | $F_{1, 96}$ = 1,240 (***) | $F_{1, 96}$ = 2,065 (***) | $F_{1, 96}$ = 2,532 (***) | $F_{1, 96}$ = 14.5 (ns) |
| Aphid species (S) | $F_{1, 96}$ = 408 (***) | $F_{1, 96}$ = 625 (***) | $F_{1, 96}$ = 692 (***) | $F_{1, 96}$ = 818 (***) | $F_{1, 96}$ = 8.2 (ns) |
| Number of aphids (A) | $F_{2, 96}$ = 545 (***) | $F_{2, 96}$ = 432 (***) | $F_{2, 96}$ = 998 (***) | $F_{2, 96}$ = 1,056 (***) | $F_{2, 96}$ = 6.4 (ns) |
| Infestation time (T) | $F_{5, 96}$ = 1,247 (***) | $F_{5, 96}$ = 780 (***) | $F_{5, 96}$ = 1,520 (***) | $F_{5, 96}$ = 1,745 (***) | $F_{5, 96}$ = 11.0 (ns) |
| G × S | $F_{1, 96}$ = 438 (***) | $F_{1, 96}$ = 523 (***) | $F_{1, 96}$ = 615 (***) | $F_{1, 96}$ = 729 (***) | $F_{1, 96}$ = 8.4 (ns) |
| S × A | $F_{2, 96}$ = 245 (**) | $F_{2, 96}$ = 296 (**) | $F_{2, 96}$ = 355 (***) | $F_{2, 96}$ = 318 (***) | $F_{2, 96}$ = 9.5 (ns) |
| G × A | $F_{2, 96}$ = 206 (**) | $F_{2, 96}$ = 362 (***) | $F_{2, 96}$ = 604 (***) | $F_{2, 96}$ = 643 (***) | $F_{2, 96}$ = 3.5 (ns) |
| S × T | $F_{5, 96}$ = 110 (**) | $F_{5, 96}$ = 154 (**) | $F_{5, 96}$ = 490 (***) | $F_{5, 96}$ = 378 (***) | $F_{5, 96}$ = 6.2 (ns) |
| G × T | $F_{5, 96}$ = 215 (**) | $F_{5, 96}$ = 376 (***) | $F_{5, 96}$ = 512 (***) | $F_{5, 96}$ = 740 (***) | $F_{5, 96}$ = 4.7 (ns) |
| A × T | $F_{10, 96}$ = 408 (***) | $F_{10, 96}$ = 257 (**) | $F_{10, 96}$ = 705 (***) | $F_{10, 96}$ = 764 (***) | $F_{10, 96}$ = 2.0 (ns) |
| G × S × A | $F_{2, 96}$ = 95 (*) | $F_{2, 96}$ = 145 (**) | $F_{2, 96}$ = 186 (**) | $F_{2, 96}$ = 316 (***) | $F_{2, 96}$ = 2.5 (ns) |
| G × S × T | $F_{5, 96}$ = 80 (*) | $F_{5, 96}$ = 105 (*) | $F_{5, 96}$ = 248 (**) | $F_{5, 96}$ = 291 (***) | $F_{5, 96}$ = 1.8 (ns) |
| S × A × T | $F_{10, 96}$ = 52 (*) | $F_{10, 96}$ = 74 (*) | $F_{10, 96}$ = 121 (**) | $F_{10, 96}$ = 205 (**) | $F_{10, 96}$ = 1.6 (ns) |
| G × A × T | $F_{10, 96}$ = 35 (*) | $F_{10, 96}$ = 92 (*) | $F_{10, 96}$ = 165 (**) | $F_{10, 96}$ = 180 (**) | $F_{10, 96}$ = 0.9 (ns) |
| G × S × A × T | $F_{10, 96}$ = 4 (ns) | $F_{10, 96}$ = 1 (ns) | $F_{10, 96}$ = 43 (*) | $F_{10, 96}$ = 62 (*) | $F_{10, 96}$ = 0.3 (ns) |

(*) $p<0.05$; (**) $p<0.01$; (***) $p<0.001$; (ns) – non-significant. Variables: i) maize genotype – Waza and Złota Karłowa; ii) aphid species – *R. padi* and *M. dirhodum*; iii) number of aphids – 0, 30 and 60 females of *R. padi* or *M. dirhodum* per plant; iv) infestation time – 0, 3, 6, 24, 48 and 96 h.

**Table S2.** Factorial ANOVA results of the tested parameters (maize genotype, aphid species, number of aphids, infestation time) and the interactions on the relative expression of the thioredoxin reductase genes (*Ftr1*, *Trxr2*), and the total activity of thioredoxin reductase (TrxR) in the maize seedlings.

| **Tested Effects and Interactions** | ***Ftr1*** | ***Trxr2*** | **TrxR** |
|---|---|---|---|
| Maize genotype (G) | $F_{1, 96}$ = 1,875 (***) | $F_{1, 96}$ = 2,150 (***) | $F_{1, 96}$ = 3,025 (***) |
| Aphid species (S) | $F_{1, 96}$ = 1,470 (***) | $F_{1, 96}$ = 1,641 (***) | $F_{1, 96}$ = 1,220 (***) |
| Number of aphids (A) | $F_{2, 96}$ = 663 (***) | $F_{2, 96}$ = 858 (***) | $F_{2, 96}$ = 984 (***) |
| Infestation time (T) | $F_{5, 96}$ = 1,584 (***) | $F_{5, 96}$ = 1,105 (***) | $F_{5, 96}$ = 2,513 (***) |
| G × S | $F_{1, 96}$ = 1,255 (***) | $F_{1, 96}$ = 986 (***) | $F_{1, 96}$ = 1,108 (***) |
| S × A | $F_{2, 96}$ = 652 (***) | $F_{2, 96}$ = 905 (***) | $F_{2, 96}$ = 615 (***) |
| G × A | $F_{2, 96}$ = 470 (***) | $F_{2, 96}$ = 743 (***) | $F_{2, 96}$ = 1024 (***) |
| S × T | $F_{5, 96}$ = 215 (**) | $F_{5, 96}$ = 560 (***) | $F_{5, 96}$ = 522 (***) |
| G × T | $F_{5, 96}$ = 284 (**) | $F_{5, 96}$ = 637 (***) | $F_{5, 96}$ = 712 (***) |
| A × T | $F_{10, 96}$ = 142 (**) | $F_{10, 96}$ = 415 (***) | $F_{10, 96}$ = 435 (***) |
| G × S × A | $F_{2, 96}$ = 115 (*) | $F_{2, 96}$ = 187 (**) | $F_{2, 96}$ = 218 (**) |
| G × S × T | $F_{5, 96}$ = 90 (*) | $F_{5, 96}$ = 151 (**) | $F_{5, 96}$ = 180 (**) |
| S × A × T | $F_{10, 96}$ = 63 (*) | $F_{10, 96}$ = 127 (**) | $F_{10, 96}$ = 92 (*) |
| G × A × T | $F_{10, 96}$ = 74 (*) | $F_{10, 96}$ = 65 (*) | $F_{10, 96}$ = 140 (**) |
| G × S × A × T | $F_{10, 96}$ = 1.6 (ns) | $F_{10, 96}$ = 0.8 (ns) | $F_{10, 96}$ = 45 (*) |

(*) $p<0.05$; (**) $p<0.01$; (***) $p<0.001$; (ns) – non-significant. Variables: i) maize genotype – Waza and Złota Karłowa; ii) aphid species – *R. padi* and *M. dirhodum*; iii) number of aphids – 0, 30 and 60 females of *R. padi* or *M. dirhodum* per plant; iv) infestation time – 0, 3, 6, 24, 48 and 96 h.

**Table S3.** List of primers and *TaqMan* fluorescent probe for real-time qRT-PCR quantification of *Ftr1* maize gene.

| Target Gene | *Genbank* Accession no. (Reference Sequence) | *GenBank* Gene ID | Sequences of Primers and *TaqMan* Fluorescent Probe |
|---|---|---|---|
| *Ftr1* (encoding ferredoxin-thioredoxin reductase 1) | X73549.1 (NM_001305869.1) | LOC542532 | F: CCGCTCCAACACTTTCTTC<br>R: TGGCATTCCTTCCTCTCAC<br>P: 5′-FAM-CGACAAGACAGTCACTGCCGTCGTCATCAA-BBQ-3′ |

Sequences of primers and *TaqMan* fluorescent probe were designed using *GenScript Real-time PCR (TaqMan) Primer Design* software (https://www.genscript.com/ssl-bin/app/primer; accessed September 02, 2019). *Custom TaqMan Gene Expression Assay* for maize *Ftr1* gene was purchased from Thermo Fisher Scientific Inc., Waltham, Massachusetts, USA (www.thermofisher.com). F – forward primer; R – reverse primer; P – *TaqMan* fluorescent probe.
